# Home delivery among antenatal care booked women in their last pregnancy and associated factors: community-based cross sectional study in Debremarkos town, North West Ethiopia, January 2016

**DOI:** 10.1186/s12884-017-1409-2

**Published:** 2017-07-14

**Authors:** Habtamu Kebebe Kasaye, Zerfu Mulaw Endale, Temesgen Worku Gudayu, Melese Siyoum Desta

**Affiliations:** 1 Midwifery Department, College of Medical and Health Sciences, Wollega University, P.O. Box 395, Nekemte, Ethiopia; 2 Midwifery Department, College of Medicine and Health Sciences, University of Gondar, P.O. Box 196, Gondar, Ethiopia; 3 Midwifery Department, College of Medicine and Health Sciences, Hawassa University, P.O. Box 1560, Hawassa, Ethiopia

**Keywords:** Antenatal care, Home delivery, Institutional delivery, Debremarkos, Ethiopia

## Abstract

**Background:**

In Ethiopia, nearly half of the mothers who were booked for antenatal care, who supposed to have institutional delivery, gave home delivery nationally. Home delivery accounts majority while few of childbirth were attended by the skilled provider in Amhara regional state. This study aimed to determine the proportion of home delivery and associated factors among antenatal care booked women who gave childbirth in the past 1 year in Debremarkos Town, Northwest Ethiopia.

**Methods:**

A community-based Cross sectional study was conducted from January 1st_−_ 25th 2016. Epi Info version 7 was used to determine a total sample size of 518 and simple random sampling procedure was employed. Data was collected through an interview by using pretested structured questionnaire. Data were entered into Epi Info version 7, cleaned and exported to SPSS version 21 for analysis. A *p*-value less than or equals to 0.05 at 95% Confidence Intervals of odds ratio were taken as significance level in the multivariable model.

**Results:**

A total of 127 (25.3%) women gave childbirth at home. Un-attending formal education (Adjusted Odds Ratio = 7.56, 95% CI: [3.28, 17.44]), absence of health facility within 30 min distance (AOR = 3.41, 95% CI: [1.42, 8.20]), not exposed to media (AOR = 4.46, 95% CI: [2.09, 9.49]), Unplanned pregnancy (AOR = 3.47, 95% CI [1.82, 6.61]), attending ANC at health post (AOR = 5.45, 95% CI: (1.21, 24.49) and health center (AOR = 2.74, 95% CI [1.29, 5.82]), perceived privacy during ANC (AOR = 3.69[1.25, 10.91]) and less than four times ANC visit (AOR = 5.04, 95% CI (2.30, 11.04]) were significantly associated with home delivery.

**Conclusions:**

Home delivery in this study was found to be low. Educational level, media exposure, geographic access to a health facility, Unplanned pregnancy, an institution where ANC was booked, perceived privacy during ANC and number of ANC visit were found to be determinants of home delivery. Health institutions, health professionals, policy makers, community leaders and all concerned with the planning and implementation of maternity care in Ethiopia need to consider these associations in implementing services and providing care, for pregnant women.

**Electronic supplementary material:**

The online version of this article (doi:10.1186/s12884-017-1409-2) contains supplementary material, which is available to authorized users.

## Background

Antenatal care is complex of interventions that a pregnant woman receives from related health care services to prevent, identify and treat complications and as well help a lady approach pregnancy and birth as positive experiences [1]. Antenatal care, skilled personnel attended delivery and postnatal care services have a crucial role in the reduction of both maternal and perinatal mortality, also they were indicators of progress toward achievement of fifth MDGs and currently also included under third SDGs. Levels of antenatal care have increased in many parts of the world, though only 46% of low-income countries benefited from attended delivery. Skilled attendant at delivery is advocated as a single most important factor to prevent maternal mortality [2, 3].

Every day over 800 maternal deaths occur worldwide, and 99% of this death is occurring in developing countries secondary to preventable causes, making maternal mortality the health statistic with the largest inequality between developed and developing countries [4]. These high maternal mortalities occur due to various direct and indirect causes, which indicate poor-quality maternal health care [5–7]. Since the launch of the motherhood initiatives, maternal mortality was taken as social injustice and health disadvantages. This consensus raised the attention and commitment of providing accessible and high-quality maternity care, even though the problem still needs more attention [8].

Although safe motherhood service has been on implementation for about three decades, overall global maternal mortality ratio was decreased from 422 in 1980 to 251 in 2008 and to 210 per 100,000 live birth in 2013 [9, 10], yet average annual reduction in Africa (1.6%) and sub-Saharan African (SSA) (1.7%) region was slowest of all other regions [11, 12]. In SSA, the burden of low-skilled birth attendant has been significant. In this region, 70% of births from poorest women occur at home and among this 56% of them were unattended [13].

Ethiopia is also among countries from which high maternal mortality has been occurring. Joint report of the maternal mortality estimates from WHO and UNICEF identified a reduction of maternal mortality to 420 per 100,000 live birth with a very wide confidence interval from 240 to 720. Antenatal care coverage at least one time and at least four times remain low even though there were encouraging improvement from 27 and 10% in 2000 to 42 and 19.1% in 2011 consecutively. The 2014 report of Central Statistical Agency revealed as Antenatal Care coverage reached 57.2% (at least one time) and 31.6% (at least four times), while the proportion of births attended by skilled health personnel improving slowly from 10% in 2011 and 14.5 in 2014 [5, 14, 15].

This discrepancy between ANC coverage and the skilled attendant at birth clearly indicates whatever women get ANC services, enormously they prefer or forced to choice home or unattended delivery. Comparative descriptive analysis demonstrated high gap between the proportion of antenatal care utilization and institutional delivery by the same individuals, which ranges 27–95% verses 4–45% [3].

The burden of home delivery, mainly that of unattended delivery is not only limited to a maternal health problem, rather it also ends up with perinatal and neonatal morbidity and mortality. Perinatal mortality found to be 21% higher in home delivery as compared to institutional delivery in SSA [16]. The opposite is true in developed country, there was no significant difference between home delivery and hospital delivery regarding perinatal or neonatal mortality, where midwives provide both hospital and home care [17].

Various reviews from a different part of the world reported factors affecting utilization of ANC service and place of delivery [18], but there was no adequate literature which answers about the reason that ANC booked women prefer home delivery or forced to do so in the study area as well in the country. This study was aimed to determine the proportion of home delivery and tried to explore factors determining home delivery among antenatal care booked women.

## Methods

### Study setting and populations

A community-based quantitative cross sectional study was conducted from January 1 to 25th, 2016 in Debremarkos town. Debremarkos town is located at 299 km Northwest of Addis Ababa and 265killometrs Southeast of the Regional capital city, Bahir Dar and it is the capital city of the East Gojjam Zone. It is found in Guzamin district and divided into seven urban kebeles (smallest administrative level) which having two rural *goxs*. Based on the 2007 national census, Guzamin district has a total population of 270,578 and Debremarkos town has a total population size of 62,469 of which 47.9% were male and 52.1% were females and by 2015/16 it was projected to be 107,234 [19].

Participants of this study were selected randomly from women who were booked for antenatal care and gave childbirth, irrespective of the outcome, within 1 year prior to January 2016. Limiting the participants to only 1-year period was made to minimize potential recall bias.

### Study variables

Place of delivery, specifically home delivery is outcome variable, while others like sociodemographic, programmatic related, women’s perceptions of quality of care during antenatal care, knowledge of the women and obstetric variables are explanatory variables included in the study. Women who answered more than the mean of knowledge questions about danger sign during pregnancy and delivery, services given during pregnancy and deliveries, importance of the services and complications during pregnancy and delivery were considered as knowledgeable while those who answered less than mean were taken as don’t know.

### Sample size and sampling procedures

A sample size of 518 was calculated using Epi Info Stat Calc version 7 population survey by taking assumptions of population size <10,000, 95% confidence interval (0.05 type one error), Expected proportion of home delivery (68.4%) from previous study in Amhara region [20], confidence limit (margin of error) 4 and 5% nonresponse rate. A total of 3265 women who had booked for ANC and gave childbirth within 1 year since January 2015 were identified by census all over 7 kebeles of the town. Then each household having those mothers were coded. Simple Random Sampling was employed using computer generated random number to identify 518 samples after developing frame having a list of individual’s house number which was given during census. Finally identified study participants were interviewed by home to home visit.

### Data collection tools and procedures

Data were collected using pretested, structured and semi-structured interviewer-administered questionnaire in the Amharic language which contains six parts. The questionnaire was first developed in English and then translated into local language Amharic by researchers. Then it was translated back to English by other people who are proficient in both languages to maintain the consistency and content of the questionnaire.

Data was collected by seven graduating class Bachelor Science Extension Midwifery students from Debremarkos University and two Bachelor Science Midwives from Debremarkos Referral Hospital were assigned to supervise the data collection process. Training was provided for 2 days for data collectors and supervisors regarding the objectives of the study, data collection method, and tools, informed consent means and significance of the study. During data collection, each data collectors was supervised for any difficulties and direction and necessary correction was provided. Collected questionnaires were checked for completeness and on spot corrective measure was taken both by data collectors and supervisors. Daily meeting was conducted between data collectors, supervisors and principal investigator for discussion regarding presenting difficulties and to assess the progress of data collection.

### Data processing and analysis

All collected questionnaires were rechecked for completeness and coded. Then these data were entered and cleaned using Epi Info 7 software and exported to SPSS version 21 for analysis. Bivariable and multivariable logistic regression were employed to identify an association between home delivery and other independent variables. All variables significantly associated with home delivery at less than or equals to 0.2 *p*-values in the bivariable logistic regression model were fitted into the multivariable logistic regression model to control the effect of confounding variables.

Multivariable analysis using backward stepwise logistic regression technique was done to evaluate the independent effect of each covariate on home delivery by controlling the effect of others. Variable having *P*-value less than or equals to 0.05 in the multivariable logistic regression analysis was considered as determinant factors for home delivery. The adjusted odd ratios with the 95%Confidence Intervals were reported. Before the actual logistic regression analysis was done, the necessary assumption of logistic regression model was checked by using Hosmer-Lemeshow test of goodness of fit which has a chi-square distribution. For further analysis, descriptive statistics like frequencies and cross tabulation were performed. Tables and bar chart were used to present the findings of the study.

The whole work of this study adhered to STROBE guidelines for the cross sectional/observational study.

## Result

A total of 502 women who had booked for antenatal care visit for their last pregnancy and gave birth within 1 year prior to the survey were interviewed which makes response rate of 96.9%, while the remaining 16 selected samples were not present at home during data collection and considered as none respondents after three continuative re-visit.

### Sociodemographic Characteristics of participants

About 421 (83.9%) of the participants were urban in residency while the remaining were from rural goxs of urban kebeles. Their age ranges from 18 to 50 with a median age of 30 and Interquartile range ± 8. The majority of the participants belongs to Amhara ethnicity 426 (84.90%) and Ethiopian Orthodox tewahido religion 481 (95.85%). More than half of the respondents who reported to have formal education were secondary school and above 262 (52.2%). Regarding marital status majority of them were married 444 (88.4%) and 218 (43.4%) of the participant women were a housewife and 133 (26.5%) of them were a government employee in occupation. 333 (71.8%) of husbands/partners’ of the respondents were educated to secondary and above while 201 (43%) of them were a government employee in occupation.

Around one-third 170 (33.9%) of the respondents’ family size was three and fewer persons. Nearly one fourth 130 (25.9%) of the household income was above 4000 Ethiopian birrs per month, while 132 (30.2%) of them got ≤2000 Ethiopian birr per month.

The majority of the respondents 442 (88%) had geographic access to a health facility in the village, among these 214 (48.4%) could be reached within 5 min by vehicle. About two-third of the respondents 332 (66.1%) were exposed to at least one means of media, while the remaining were not exposed (see Table 1).

**Table 1:** Sociodemographic Characteristics of study participants in Debremarkos town, North West Ethiopia, January 2016 (*n* = 502)

Characteristics	Frequency	Percentage
Maternal residence		
Urban	421	83.9
Rural	81	16.1
Maternal age in year (Median = 30, IQR = 8)		
15–19	4	0.8
20–24	54	10.7
25–29	158	31.5
30–34	145	28.9
35 and above	141	28.1
Maternal Marital Status		
Single	27	5.4
Married	444	88.4
Divorced/Widowed	31	6.2
Maternal Ethnicity		
Amhara	481	95.8
Oromo	17	3.4
Others ^a^	4	0.8
Maternal Religion		
Orthodox	426	84.9
Protestant	20	4.0
Muslim	56	11.1
Maternal Educational Status		
No Formal education	118	23.5
Primary education	122	24.3
Secondary and above	262	52.2
Maternal Occupation		
Housewife	218	43.4
Gov’t employee	133	26.5
NGO employee	23	4.6
Private Business	103	20.5
Student	25	5.0
Husband/ Partner Educational status (*n* = 464)		
No formal education	109	23.5
Primary Education	22	4.7
Secondary and above	333	71.8
Husband/Partner Occupation(*n* = 467)		
Farmer	82	17.6
Gov’t employee	201	43.0
NGO employee	52	11.1
Private Business	120	25.7
Others^b^	12	2.6
House Hold Income per month in Birr		
≤2000	152	30.3
2001–4000	220	43.8
≥4000	130	25.9
Presence of Health Facility in the village within 30 min distance on foot		
No	60	12.0
Yes	442	88.0
Time to reach Health facility by vehicle/min (Mean ± SD = 8.60 ± 5.75)(*n* = 442)		
≤5 min	214	48.4
5–10 min	147	33.3
>10 min	81	18.3
Family Size		
≤3 person	170	33.9
4–5 person	204	40.6
≥6 person	128	25.5
Media Exposure		
Exposed to at least one source of media	332	66.1
Not exposed to any media source	170	33.9

^a^Tigraway, Gurage

^b^Daily laborer & No occupation

### Past Obstetrics Characteristics of the respondents

Regarding age at first marriage, 114 (23.7%) of the respondents were married before the age of 18. Two hundred eighty-two (56.2%) of the responding women were first pregnant while their age was between 19 and 24. About 74 (14.7%) of the respondents were delivered five and more times. 143 (28.5%) of the respondents had experience of prolonged labor before the index delivery and nearly half 178 (51.6%) of the previous deliveries were took place at home. About one-third 119 (33.8%) of individuals had experienced at least one of bad obstetric history before their last pregnancy.

Regarding their last pregnancy, 382 (76.1%) women reported that their last pregnancy was planned and majority 347 (69.1%) of them attended ANC at the health center. 401 (79.9%) of the respondents were started ANC before 16 weeks of gestational age, and only 168 (33.5%) of the respondents were attended four and above visits of antenatal care (see Table 2).

**Table 2:** Past Obstetrics Characteristics of study participants in Debremarkos town, North West Ethiopia, January 2016. (*n* = 502)

Characteristics	Frequency	Percentage
Age at First Marriage (Mean ± SD = 21.03 ± 3.43)		
<18 Years	114	23.7
≥18 Years	368	76.3
Age at first pregnancy (Mean ± SD = 23.45 ± 4.07)		
≤18 years	52	10.4
19–24 years	282	56.2
25–30 years	136	27.1
Above30years	32	6.4
Number of pregnancy		
I	153	30.5
II–IV	249	49.6
V	100	19.9
Number of delivery		
≤4	428	85.3
≥5	74	14.7
Experience of Prolonged labor greater than 12 h previously		
No	359	71.5
Yes	143	28.5
Previous place of delivery (*n = 345*)		
Health facility	167	48.4
Home	178	51.6
Experience of Bad Obstetrics History (*n = 352*)		
No	233	66.2
Yes	119	33.8
Pregnancy Plan		
Unplanned	120	23.9
Planned	382	76.1
Place where ANC attended		
Health Post	26	5.2
Health Center	347	69.1
Hospital	125	24.9
Others^a^	4	0.8
GA at first ANC visit		
Before 16 weeks	401	79.9
16–28 weeks	94	18.7
After 28 weeks	7	1.4
Number of ANC visit completed		
One	18	3.6
Two and Three	316	62.9
Four and above	168	33.5

^a^Marrie stops, EFGA

### Perceived characteristics of ANC and knowledge of the participants

The majority of the respondents 462 (92.0%) and 492 (98.0%) reported that they received an explanation from providers about their health condition and what to expect during labor and delivery respectively. Similarly, a great deal of responding women reported that they received respect and privacy from the service provider during antenatal care.

Concerning behavior of the care providers about 226 (45%) of them revealed as they have good behavior and only 19 (3.8%) of respondents reported quality of ANC as poor while the rest said it was satisfactory and good. About 319 (63.5%) of the respondents received ANC service in average within 30 min of their arrival to the health facility. Almost all of the respondents reported as they were advised on the importance of institutional delivery, danger signs during pregnancy and delivery during ANC 444 (88.4%) and the rest during a home visit by HEW.

Knowledge of the participants refers to the participants’ understanding of the advice they received from the health care providers during ANC or home visit. Based on this score they acquired from the total knowledge assessing questions, the respondents classified as knowledgeable or not, taking the mean score as differentiation for knowledge. Among 33 knowledge questions respondents were asked on danger sign, services, complications during pregnancy and delivery 214 (42.6%) of the respondents scored correct answers for the questions above mean (which was 18) of the overall correct response, while 288 (57.4%) of them scored less than mean of knowledge questions score. Concerning ANC service providers 316 (62.9%) received from Midwives and 59 (11.8%) of them from HEW. Among these 424 (84.5%) of the respondents received high-quality ANC from skilled health care providers while the remaining received comparably low-quality ANC from HEW (see Table 3).

**Table 3:** Counseling and communication with health providers during ANC, and perceived behavior of health care providers in Debremarkos town, North West Ethiopia, January 2016 (*N* = 502)

Characteristics	Number	Percent
Providers explained your condition in an understandable way		
No	40	8.0
Yes	462	92.0
Providers explained what to expect during labor and delivery		
No	10	2.0
Yes	492	98.0
Providers listened to your questions or concerns		
No	63	12.5
Yes	439	87.5
Did the providers respect you		
No	30	6.0
Yes	472	94.0
Privacy protected during the examinations		
No	51	10.2
Yes	451	89.8
Perceived Behavior of ANC service providers		
Very Good	208	41.4
Good	226	45.0
Fair	55	11.0
Bad	13	2.6
Time taken to get ANC		
30 min and less	319	63.5
31–59 min	89	17.7
60 min and more	94	18.8
Perceived quality of ANC		
Good	383	76.3
Satisfactory	100	19.9
Poor	19	3.8
Timing of advice on ID & danger sign during pregnancy and delivery		
During ANC	444	88.4
During home visit by HEW	57	11.4
Fear to be exposed during delivery		
Yes	145	28.9
No	350	69.7
Others^a^	7	1.4
ANC providers by profession		
Midwives	316	62.9
Nurses	85	16.9
Medical Doctors	18	3.6
Public Health Officers	6	1.2
HEW	59	11.8
Don’t know	18	3.6
Knowledge of Participants		
Don’t know	288	57.4
Knows	214	42.6

^a^Missing

### Maternal decision power on health care

409 (86.1%) individuals reported that they have discussed with their husband or partner about the place of delivery and majority of them 461 (91.8%) preferred to deliver at health facility while comparably 424 (90.6%) of husbands preferred the same place. Regarding delivery attendant 16 (3.2%) of respondents and 30 (6.4%) of husbands preferred TBA and one of family members respectively. Nearly three fourth 383 (76.3%) of the final decision regarding the place of delivery was given by both respondent and her husband, while 93 (18.5%) of the respondents gave by themselves on the place of delivery. 54 (10.7%) of a financial decision for costs related to pregnancy and delivery were decided only by their husband (see Table 4).

**Table 4:** Discussion habit and Maternal decision power on the place of delivery in Debremarkos town, North West Ethiopia, January 2016 (*N* = 502)

Characteristics	Number	Percent
Discussed with your husband/partner on place of delivery?(*N* = 475)		
No	66	13.9
Yes	409	86.1
Where did you choice to deliver		
Home	35	7.0
Health Facility	461	91.8
Others^a^	6	1.2
Where did your husband prefer you to deliver (468)		
Home	35	7.5
Health Facility	424	90.6
Others^a^	9	1.9
Whom did you prefer to attend your delivery		
Skilled Health Professional	465	92.6
Trained TBA	16	3.2
One of my family member	21	4.2
Whom did your husband choice to attend your delivery		
Skilled Health Professionals	432	91.9
Trained TBA	8	1.7
One of my family member	30	6.4
Where did other community members prefer to you to deliver		
Home	33	6.6
Health Facility	469	93.4
Where did other family members prefer you to deliver		
Home	75	14.9
Health Facility	425	84.7
Others^a^	2	0.4
Who gave final decision on place of delivery		
Me	93	18.5
My husband	16	*3.2*
Both me and my husband	383	76.3
My Parents	10	2.0
Who decide on costs related to pregnancy and delivery		
Me	41	8.2
My husband	54	10.7
Both me and my husband	385	76.7
My Parents	22	4.4

^a^Private clinic

### Home delivery and reasons given

From all respondents, 127 (25.3%, 95% CI [21.6, 28.7]) of them gave childbirth at home while 375 (74.7%) of the respondents delivered at a health facility. Majority 107 (84.3%) of home deliveries took place at women’s own home.

Of the total home deliveries, 74 (58.3%) of them were assisted by women’s relatives, nearly one fourth 34 (26.8%) of these deliveries were not attended and the remaining 19 (14.9%) of deliveries were assisted by respondents’ mother, husband, and TBAs (see Table 5).

**Table 5:** Ideas given on institutional delivery and actual place of delivery in Debremarkos town, North West Ethiopia, January 2016 (*n* = 502)

Characteristics	Number	Percent
Maternal idea on institutional delivery		
It is Necessary	498	99.2
Not Necessary	4	0.8
Family’s idea on necessity of institutional delivery		
It is Necessary	433	86.2
Not Necessary	29	5.8
Not Customary	40	8.0
Place of Current Delivery		
Health Facility	375	74.7
Home	127	25.3
Place at home delivery specifically (*N* = 127)		
My own home	107	84.3
My parents’ home	16	16.6
Religious home	4	3.1
Delivery Attendant for home delivery (*N* = 127)		
Relatives	74	58.3
No one	34	26.8
Others ^a^	19	14.9
Which Health Institution for Health facility delivery (*N* = 375)		
Public Hospital	165	44.0
Health Center	198	52.8
Private and NGO clinics	12	*3.2*

^a^My mother, my husband, TBAs

**Fig. 1:**
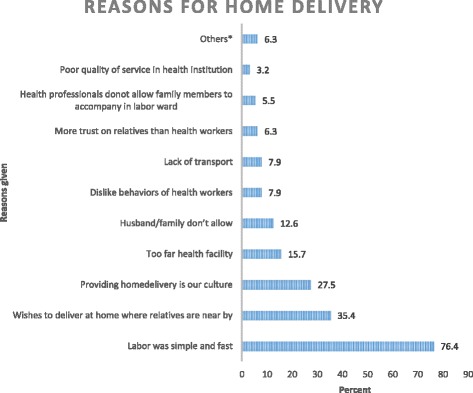
Reasons given by respondents on home delivery in Debremarkos, North West Ethiopia, January 2016 (*n* = 127). *Fear of HIV infection, health facility was close and I was alone

Responding women gave a variety of reasons for providing home delivery. Among these ‘*labor was simple and fast’ 97 (76.4%)*, ‘*wishes to deliver at home where relatives are nearby’* 45 (35.4%) and ‘*providing home delivery is our culture*’ 35 (27.6%) were leading (see Fig. 1).

### Determinants of home deliveries

In Bivariable logistic regression marital status, maternal occupation, maternal residence, maternal educational level, presence of health facility in the village within 30 min, media exposure, family size, maternal age at first pregnancy, number of delivery, experience of prolonged labor, planned pregnancy, facility at which ANC attended, number of ANC visit completed, GA at first ANC visit, maternal knowledge on maternity service, perceived respect during ANC, perceived privacy during ANC, time to wait to get ANC in minutes, decision maker on cost needed for referral and decision maker on place of delivery were the factors found to be statistically significantly associated with home delivery.

All variables associated with home delivery in bi-variable analysis at 0.2 *P*-value were fitted to multivariable logistic regression model in order to control confounding factors. Accordingly un-attending formal education (AOR = 7.56, 95% CI: [3.28, 17.44]), absence of health facility within 30 min distance (AOR = 3.41, 95% CI: [1.42, 8.20]), none exposed to media (AOR = 4.46, 95% CI: [2.09, 9.49]), unplanned pregnancy (AOR = 3.47, 95% CI [1.82, 6.61]), attending ANC at health post (AOR = 5.45, 95% CI: (1.21, 24.49), health center (AOR = 2.74, 95% CI [1.29, 5.82]), perceived privacy during ANC (AOR = 3.69[1.25, 10.91]) and less than four times ANC visit (AOR = 5.04, 95% CI (2.30, 11.04]) were factors found independently associated with home delivery at *p*-value of less than or equals to 0.05 (see Table 6).

**Table 6:** Determinants of Home delivery from backward stepwise (Wald) logistic regression in Debremarkos, North West Ethiopia, January 2016

Variables	Place of delivery		Crude OR OR (95% CI)	Adjusted OR OR (95% CI)	*P*-Value
	Home (%)	HI (%)			
Marital status					
Single	12 (44.4)	15 (55.6)	2.5 (1.17, 5.7)	*	
Divorced/Widowed	10 (32.3)	21 (67.7)	1.5 (0.7, 3.4)		
Married	105 (23.6)	339 (76.4)	1		
Maternal education level					
No Formal Education	78 (66.1)	40 (33.9)	16.97 (9.77, 29.45)	*7.56 (3.28, 17.44)*	*<0.001*
Primary school	22 (18)	100 (82)	1.9 (1.04, 3.52)	*	
Secondary and above	27 (10.3)	235 (89.7)	1	*1*	
Maternal Occupation					
NGO/Gov’t Employee	12 (7.7)	144 (92.3)	0.13 (0.07, 0.26)	*	
Private business	23 (23.3)	80 (77.7)	0.47 (0.27, 0.81)		
Student	10 (40)	15 (60)	1.10 (0.47, 2.57)		
Housewife	82 (37.6)	136 (62.4)	*1*		
Maternal Residency					
Rural	49 (60.5)	32 (39.5)	6.73 (4.05, 11.20)	*	
Urban	78 (18.5)	343 (81.5)	1		
Presence of Health facility within 30’					
No	38 (63.3)	22 (36.7)	6.85 (3.86, 2.17)	*3.41 (1.42, 8.20)*	*0.006*
Yes	89 (20.1)	353 (79.9)	1	*1*	
Family size in person					
≤ three	36 (21.2)	134 (78.8)	0.28 (0.17, 0.46)	*	
4–5	28 (13.7)	176 (86.3)	0.16 (0.09, 0.28)		
6 and above	63 (49.2)	65 (50.8)	1		
Media Exposure					
Not exposed	95 (55.9)	75 (44.1)	11.87 (7.40, 19.10)	*4.46 (2.09, 9.49)*	*<0.001*
Exposed	32 (9.6)	300 (90.4)	1	*1*	
Maternal age at 1st pregnancy					
<18 years	31 (59.6)	21 (40.4)	5.44 (2.99, 9.90)	*	
≥18 years	96 (21.3)	354 (78.7)	1		
Number of Parity/delivery					
Four and less	81 (18.9)	347 (81.1)	0.14 (0.08, 0.24)	*	
Five and above	46 (62.2)	28 (37.8)	1		
Experience of Prolonged Labor greater than 12 h					
No	82 (22.8)	277 (77.2)	0.64 (0.42, 0.99)	*	
Yes	45 (31.5)	98 (68.5)	1		
Perceived respect during ANC					
No	20 (66.7)	10 (33.3)	6.68 (4.07, 11.4)	*	
Yes	107 (22.7)	365 (77.3)	1		
Perceived privacy during ANC					
No	32 (62.7)	19 (37.3)	6.31 (4.23, 9.4)	*3.69 (1.25, 10.91)*	*0.018*
Yes	95 (21.1)	356 (78.9)	1		
Time to wait to get ANC in minutes					
30 and less	95 (29.8)	224 (70.2)	2.42 (1.6, 3.6)	*	
31–59	18 (20.2)	71 (79.8)	1.44 (0.88, 2.4)		
60 and more	14 (14.9)	80 (85.1)	1		
Pregnancy Plan					
Unplanned	64 (53.3)	56 (46.7)	5.78 (3.69, 9.06)	*3.47 (1.82, 6.61)*	*<0.001*
Planned	63 (16.5)	319 (83.5)	1	*1*	
Place where ANC attended					
Health Post	14 (53.8)	12 (46.2)	8.23 (3.24, 20.9)	*5.45 (1.21, 24.49)*	*0.027*
Health Center	97 (28)	250 (72)	2.74 (1.54, 4.86)	*2.74 (1.29, 5.82)*	*0.008*
Public Hospital	16 (12.4)	113 (87.6)	1	*1*	
GA at first ANC Visit					
≤16 weeks	80 (20.0)	321 (80.0)	1	*	
>16 weeks	47 (46.5)	54 (53.5)	3.49 (2.20, 5.54)		
Number of ANC visit completed					
One to three	113 (33.8)	221 (66.17)	5.62 (3.11, 10.17)	*5.04 (2.30, 11.04)*	*<0.001*
Four and above	14 (8.3)	154 (91.67)	1	*1*	
Maternal Knowledge on maternity service					
Don’t know	93 (39.7)	141 (60.3)	4.54 (2.91, 7.08)	*	
Knows	34 (12.7)	234 (87.3)	1		
Decision maker on cost needed for referral					
Me	20 (48.8)	21 (51.2)	4.6 (2.9, 7.1)	*	
My husband/ other members of family	41 (53.9)	35 (46.1)	5.66 (4.0, 7.9)		
Both me and my husband	66 (17.1)	319 (82.9)	1		
Decision maker on place of delivery					
Me	60 (64.5)	33 (35.5)	10.4 (7.45, 14.5)	*	
My husband/ other members of family	10 (38.5)	16 (61.5)	3.5 (2.06, 6.2)		
Both me and my husband	57 (14.9)	326 (85.1)	1		

*-Variables which were not associated in backward stepwise multivariable logistic regression model, 1-Reference category

## Discussion

This study was aimed to assess the proportion of home delivery and associated factors among ANC booked and gave birth in the last 12 months preceding survey. It is reasonable to assess proportion and associated factors of home delivery among ANC utilized individuals in an urban community where having more infrastructures in healthcare facilities than rural areas, and thus, usage of institutional delivery is expected to be high.

The result of this study revealed that 127 (25.3%, with 95% CI [21.6, 28.7]) of total respondents were given delivery at home. The proportion of home delivery in this study is lower than 2011 EDHS (76.25% National and 89.3% of Amhara region) and study done in Fogera district of Northwest Ethiopia which was 68.4% of total participants. This large difference could be due to the difference in residency, as most of the participant in those studies were residents from rural [21, 22].

The magnitude of home delivery in this study is also lower than the result of a study done 4 years back in the same setting on the preference of place of delivery in which 37.7% of them delivered at home. It is also lower than studies done in other parts of Ethiopia [22–30] which could be because of time and study subjects’ difference as these studies included individuals who did not have antenatal care follow up which is one of the most known predictors of home delivery. A lot of contributions had been made to change the awareness of women’s and communities’ on the utilization of institutional delivery which might result in this decreased home delivery than these mentioned studies. It might be due to the fact that most of the respondents and majority (86.2%) of their family believed in the necessity of institutional delivery in this study.

It is also lower than studies done in other developing countries like Kenya [31], Tanzania [32], Ghana [33], Nigeria [34], Nepal [35], this might be due to presence of a difference in the health service delivery system, socio-cultural difference, economic or study setting difference.

It is consistent with a study done in Bahir Dar city administration and another African country like Nigeria and Senegal. This might be because the majority of the respondents were from urban and ANC booked [36–38].

In another study from Nepal [39] proportion of home delivery (15%) was lower than this study, which might be because of difference in study design. The study from Nepal had a nature of follow-up in which participants were asked about their preference of place of delivery, which could have imposed them to had more institutional delivery than home.

Educational status of the responding women and husband was reported as a determinant factor to home delivery from the vast majority of studies done both in our country and abroad [21–30, 34, 40, 41]. The result of this study also supports those findings as being uneducated was significantly associated with home delivery. Mothers who did not have any formal education were seven times (AOR = 7.56, 95% CI: [3.28, 17.44]), more likely to deliver at home than those educated to secondary and above educational levels. Education is a major key strategy to enhance health care service utilization. However, if there is no universal access to education it would be the big gap between educated and not in understanding the difficulties of laboring and childbirth as well complications related to unattended delivery. Being educated increase the awareness of preventive health care services and the receptive ability for new information. Education could also enhance women autonomy which enables them to discuss and determine on the place of delivery. Due to these facts, women who did not attend any formal education ended up with home delivery than their counterparts.

Media exposure was also another determinant of home delivery with four times (AOR = 4.46, 95% CI: [2.09***,*** 9.49]) likelihood of delivering at home among not exposed group than exposed ones. This is in line with the studies done in Gondar and Holeta. This could be due to the fact that individuals whom having media exposure had the ability to attend the awareness information on the importance of institutional delivery. Media also have the ability to change attitudes women and communities used to have on home delivery, as a result individual who not exposed to any source of media might be continued having home delivery more likely than those who exposed [23, 40].

Distance to the nearest health facility is also found to be associated with home delivery which was also reported from Jimma [42]. Individuals who lack geographic access to a health facility within a 30 min distance from women’s home were three times (AOR = 3.41, 95% CI: [1.42, 8.20]) more likely to have home delivery than their counterparts. Presumably the shorter the distance to the health facility the easier it is for a woman to get there, even if she has to walk. Pregnant mother’s effort to travel on foot even when alternative traveling means is absent could be higher when the distance to a nearby health facility is shorter. Walking more kilometers is difficult in labor and it worse if the labor starts at night and very urging as most of the respondents gave it as a reason for home delivery.

Individuals who unplanned their last pregnancy had three (AOR = 3.47, 95% CI [1.82, 6.61]) times likelihood of delivering at home than those who had planned their last pregnancy. Planning to have child needs mental, psychological and financial readiness. If one planned to have a child it means that this person fitted with these necessities. Again planned pregnancy indicates the presence of support person either the husband or partner. So as the pregnancy was planned and supported the probability of seeking institutional delivery increases. As a result, the likelihood of having home delivery could increase as one had unplanned pregnancy.

Institutions, where ANC was attended, was also found to be one of the predictors of home delivery. Having ANC attendance at health post were five times (AOR = 5.45, 95 CI: [1.21, 24.49]) more likely to deliver at home than whom attended at the hospital. Similarly, women who attended ANC at health center were three times (AOR = 2.74, 95% CI [1.29, 5.82]) more likely to deliver at home than those who attended ANC at the hospital. Most of the health care providers in health center are midlevel educated and lack vast of experience, which might have helped them in counseling during antenatal care on the place of delivery. Though more investigations are needed, lack of experience, the absence of specialized health personnel in health post and health center and quality of ANC might be related to increased odds of home delivery.

Perceived lack of privacy during ANC increases the odds of home delivery four times than (AOR = 3.69, 95% CI: [1.25, 10.91]) who believed that they had been possessed privacy while ANC. It is in line with a report from Fogera district [20]. This might be because individuals who perceived a lack of privacy during ANC expect the same while they revisit health facility for childbirth. This could be also due to the fact that most of the respondents who perceived lack of privacy reported that they fear to expose their reproductive organs during delivery.

Similarly, as reported from other studies [20, 33, 39–41], increments in a number of ANC visit will result in less likelihood of delivering at home. Individuals who did not complete recommended four visits were five times (AOR = 5.04, 95% CI (2.30, 11.04]) more likely to deliver at home when compared to those who had four and more ANC visits. This might be because number of the visit will increase the chance of having more awareness on the importance of institutional delivery. It also creates good opportunity on increasing counseling mothers got from health professionals which have great ability in minimizing home delivery. In addition, mostly practiced timing of advice on birth preparedness, especially on the place of delivery is in later gestational age and with increased number of visit.

### Limitation of the study

Cross sectional nature of the study and some of the questions demanding in-depth interview and focused group discussion were simply addressed with open-ended questions due to different reasons.

## Conclusion and recommendation

The proportion of home delivery in this study among women who were booked for antenatal care and gave childbirth within 1 year prior to survey time was found to be low. Un-attending formal education, presence of health facility within 30 min distance on foot, none exposed to media, unplanned pregnancy, attending antenatal care at health post and health center, perceived lack of privacy during antenatal care and antenatal care visits less than four times were factors found to be associated with home delivery significantly.

All concerned stakeholders need to take actions to alleviate the presenting problem by increasing educational opportunity for all women, enhancing geographic access to a health facility, awareness creation on the importance of media and utilization of family planning services in order to decrease home delivery for antenatal care booked women. The quality of antenatal care at health post and health centers has to be measured as individuals who received antenatal care at these facility tends to have more home delivery. In addition, there have to be strategies in which a number of antenatal care visit could be raised.

## Additional file

Additional file 1: Questionnaire-English Version. This file presents the questionnaire used to collect data for this study. It contains separate six parts which presented in a total of nine pages including the title and an introcduction section. (DOCX 30 kb)

## Abbreviations

ANC: Antenatal care
AOR: Adjusted Odds Ratios
CI: Confidence interval
COR: Crudes Odds Ratios
CSA: Central Statistical Agency
EDHS: Ethiopian Demographic and Health survey
HIV: Human Immunodeficiency Virus
MDGs: Millennium Development Goals
OR: Odds Ratio
SDGs: Sustainable Development Goals
SSA: Sub Saharan Africa
UNFPA: United Nations Population Fund
WHO: World Health Organization
