# Supplementary material for: Home delivery among antenatal care booked women in their last pregnancy and associated factors: community-based cross sectional study in Debremarkos town, North West Ethiopia, January 2016
Source: BMC Pregnancy Childbirth. 2017 Jul 14;17:225. doi: 10.1186/s12884-017-1409-2 (PMC5512956; doi:10.1186/s12884-017-1409-2)
Supplement: Additional file 1: — Questionnaire-English Version. This file presents the questionnaire used to collect data for this study. It contains separate six parts which presented in a total of nine pages including the title and an introcduction section. (DOCX 30 kb) [file 12884_2017_1409_MOESM1_ESM.docx]

## Additional file 1: Questionnaire- English Version

**Descriptions of the material:**

This document presents the questionnaire used to collect data for research entitled with ‘Home delivery among antenatal care booked women in their last pregnancy and associated factors: community-based cross sectional study in Debremarkos town, North West Ethiopia, January 2016’. It contains separate six parts which includes Socio- demographic and economic, obstetrics characteristics of respondents, institutional care related, responding women’s knowledge, decision ability, actual place of last childbirth with its reasons.

**General Instruction for data collectors:** For the questions listed at the second column next to their sequence number, there is given alternative responses. For the responses given by participant please encircle the choice indicating their response. There are questions might be having more than one response, in that case there will be multiple responses. Some questions need to be skipped to the next questions, please don’t forget to refer the remark column.

**Part One: Respondent’s socio-demographic and economic characteristics**

| **Sr. No.** | **Questions** | **Alternative choices of response** | **Remark** |
| --- | --- | --- | --- |
|  | Age | __________years |  |
|  | Religion | 1. Ethiopian Orthodox Tewahido 2. Protestant 3. Muslim 4. Catholic 5. Adventist 6. Other(specify)____________ |  |
|  | Ethnicity | 1. Amhara 2. Tigre 3. Oromo 4. Other(specify)___________ |  |
|  | Marital status | 1. Never married 2. Married 3. Widowed 4. Divorced 5. Separated |  |
|  | Occupation | 1. House wife 2. Government employee 3. Nongovernmental employee 4. Private Business 5. Student 6. Others Specify)____________ |  |
|  | Occupation of the Husbands if married | 1. Farmer 2. Government employee 3. Nongovernmental employee 4. Student 5. Private Business 6. Others (Specify)____________ |  |
|  | Residency | - 1. Urban   2. Rural |  |
|  | House Hold Income per month in Birr for urban residency | ____________________ |  |
|  | Amount of cereal income from last year in quintal (for whom are rural in residency ) | Teff_________________-  Maize________________-  Barley________________-  Potatoes________________-  Wheats________________- |  |
|  | Number of domestic animals (for whom are rural in residency ) | Oxen________________-  Cows________________-  Sheep________________-  Goats________________-  Donkeys________________-  Hens________________-  Others________________- |  |
|  | Maternal Educational Level | 1. Don’t educated 2. Read and Write 3. Some of Primary Education 4. Completed Primary Education 5. Some of Secondary Education 6. Completed secondary education 7. Above secondary |  |
|  | Husbands’ Educational Level if married | 1. Don’t educated 2. Read and Write 3. Some of Primary Education 4. Completed Primary Education 5. Some of Secondary Education 6. Completed secondary education 7. Above secondary |  |
|  | Is there health center or hospital in your area? | 1. Yes 2. No |  |
|  | If yes how many time it take to reach HF? | __________in vehicles hours |  |
|  | How much is your family size | ___________no. of persons |  |
|  | Do you fear expose your reproductive organ during delivery? | 1. Yes 2. No |  |
|  | Exposure to media in weekly bases (**more than one choice is possible out of three possibilities**) | - 1. Reads a newspaper at least once a week   2. Watches television at least once a week   3. Listens to the radio at least once a week   4. Accesses all three media at least once a week   5. Accesses none of the three media at least once a week |  |

**Part Two: Obstetrics and Maternal health care factors**

| **Sr. No.** | **Questions** | **Alternative choices of response** | **Remark** |
| --- | --- | --- | --- |
| **201.** | Age at first marriage (at first union ) | In years--------------- |  |
| **202.** | Age at first pregnancy | In years--------------- |  |
| **203.** | Gravidity | ___________no. of pregnancy |  |
| **204** | Parity | ___________no. of parity |  |
| **205.** | Have you been experienced prolonged labor | 1. Yes 2. No |  |
| **206.** | Where did you give birth your previous last child? | 1. At my own home 2. My parents’ home 3. Health facility 4. TBA home 5. Religious home 6. Other (specify)________ |  |
| **207.** | Experience of bad obstetric history previously | 1. Cesarean section delivery 2. Abortion 3. Still birth 4. IUFD 5. Neonatal loss 6. Others, specify ___________________ 7. No obstetric complication is experienced |  |
| **208.** | Was this pregnancy planned? | 1. Yes 2. No |  |
| **209** | Where did you attended ANC? | 1. Health post 2. Health center 3. Hospital 4. Other place_______ |  |
| **210** | Number ANC visit received? | ______________ in numbers |  |
| **211** | Gestational age at first ANC visit? | ______________ in weeks |  |

**Part three: Institutional Care Related Questions: Counseling and communication during ANC**

| **Sr. No.** | **Questions** | **Alternative choices of response** | **Remark** |
| --- | --- | --- | --- |
| **301.** | Did the providers explained your health condition with terms that you able to understand? | 1. Yes 2. No |  |
| **302.** | Did the providers explained what to expect during labor and delivery? | 1. Yes 2. No |  |
| **303.** | Did the health providers listened to your questions or concerns? | 1. Yes 2. No |  |
| **304.** | Did the providers respect you? | 1. Yes 2. No |  |
| **305.** | Did the provider kept protected your privacy during the examinations? | 1. Yes 2. No |  |
| **306.** | How do you rank the behavior of health workers providing ANC services? | 1. Very good 2. Good 3. Fair 4. Bad |  |
| **307.** | How long was the time you spent in waiting to get ANC services? | _________ minute |  |
| **308.** | What do you think the quality of ANC given? | 1. Good 2. Satisfactory 3. Poor |  |
| **309.** | Did you get advice about the need to have delivery at health facilities? | 1. Yes 2. No |  |
| **310.** | When did you get the advice? | 1. During ANC visit 2. During home visit by HEW 3. During contact with TBA |  |
| **311.** | From whom did you got ANC service? | 1. Midwife 2. Nurse 3. Doctor 4. Health Officer 5. Health Extension Worker 6. Others, specify ___________________ |  |

**Part four: Knowledge related questions**

| 401 | Do you Know danger signs during pregnancy and labor? | 1. Yes 2. No |  |
| --- | --- | --- | --- |
| 402 | If “yes” what are they? **** Don’t read the choices, multiple response is possible** | 1. Vaginal bleeding 2. Persistent vomiting 3. Severe headache 4. Blurring of vision 5. Severe upper right upper quadrant abdominal pain 6. Seizure 7. Obstructed labor/ prolonged labor 8. Retained placenta 9. Others, specify ______________________ |  |
| 403 | What are the pregnancy and delivery related services given in health facilities? | 1. ANC services 2. Delivery services 3. PNC services 4. Prevention of delivery complications 5. Managing delivery complications 6. Managing health problem of the New Born |  |
| 404 | What do you think the advantages of pregnancy and delivery related services? | 1. For anticipating problems 2. For early detection of health problems 3. For appropriate management of health problems 4. For better health care to the women 5. For better care to the newborn |  |
| 405 | What complications do you know that can occur during pregnancy? | 1. Vaginal bleeding 2. Severe headache 3. Severe abdominal pain 4. Marked & fast weight gain 5. Amniotic fluid leakage 6. Absence of fetal movement |  |
| 406. | Who are do you think susceptible for pregnancy and delivery complications? | 1. Every mother including myself 2. Primi-gravida-mothers 3. Multi gravid mothers (5 and more) 4. Mothers with multiple pregnancy 5. Mothers with other medical problems |  |
| 407. | What are the complications that can occur during delivery? | 1. Severe hemorrhage 2. Retained placenta (lasting more than 30 minutes) 3. Prolonged labor (lasing more than 12 hours) 4. Loss of consciousness |  |

**Part Five: Decision Related Questions**

| 501 | Did you discuss with your partner about where to deliver during your last delivery? | 1. yes  2. No |  |
| --- | --- | --- | --- |
| 502 | What was your preference of delivery place during your last delivery? | 1. Home 2. Health facilities 3. Other places, specify-------88 |  |
| 503 | What was your husband’s preference about delivery place during your last delivery? | 1. Home 2. Health facilities 3. Other places, specify--------88 |  |
| 504 | What was your preference about attendant of delivery during your last delivery? | 1. SBA  2. TTBA  3. TBA  4. Family member or relatives  5. Others, specify---------------88 |  |
| 505 | What was your husband’s preference about attendant of delivery during your last delivery? | 1. SBA  2. TTBA  3. TBA  4. Family member or relatives  5. Others, specify-------------88 |  |
| 506 | What was the preference of other family members about place of delivery during your last delivery? | 1. Home 2. Health facilities 3. Other, specify---------------88 |  |
| 507 | What was the preference of the community about the place of delivery during your last delivery? | 1. Home 2. Health facilities 3. Other, specify----------------88 |  |
| 508 | Who was finally made decision about your delivery place during your last delivery? | 1. Myself  2. My husband  3. Both me & my husband  4. Others, specify--------------88 |  |
| 509 | Who decides on the cost related to health care/ for referral or reaching health facility? | 1. Myself  2. My husband  3. Both me & my husband  4. Others, specify--------------88 |  |

**Part six: Ideas on institutional delivery and Actual place of delivery and reasons given to Place of delivery**

| **Sr. No.** | **Questions** | **Alternative choices of response** | **Remark** |
| --- | --- | --- | --- |
| **601** | What do you think about necessity of institutional delivery? | 1. It is necessary 2. Not necessary |  |
| **602** | What do your family think about necessity of institutional delivery? | 1. They all think it is Necessary 2. Father did not think necessary 3. Family did not think necessary 4. Husband did not think necessary 5. Not customary |  |
| **603** | Whom do you prefer to attend your delivery among skilled health care provider and TBA | 1. Health care provider 2. TBA |  |
| **604.** | Where did you delivery your current child? | 1. Home 2. My own home 3. My parent’s home 4. My relatives home 5. Religious home 6. Others, specify __________________ 7. Health facility 8. Public Hospital 9. Private Clinic 10. NGO’s Clinic 11. Health center 12. Health post 13. Others, specify ___________________ | **If ‘b’ Skip to Q.**607 |
| **605.** | If your answer is “a” for the above question, why do you prefer home delivery? **Multiple answer is possible** | 1. Institutional delivery is not necessary 2. Providing home delivery is our culture 3. Institutional delivery is too costly 4. Health professionals do not allow family members to accompany in labor ward 5. Dislike behaviors of health workers at health institution 6. Too far health facility and lack of transport 7. Facility is not open 8. More trust on TBA/relatives than health workers at health institution 9. Poor quality of service in health institution 10. Labor was simple and normal 11. Wishes to deliver at home where relatives are nearby 12. Husband/ family don’t allow 13. Others, specify___________________ |  |
| **606.** | Who assisted/attend you while delivery? | 1. TBA 2. Relatives 3. No one 4. Others, specify______________________ |  |
| **607.** | If your answer is “b”, why did you want to deliver your baby in that particular place? **Multiple answer is possible** | 1. Close to where I live 2. High quality services 3. Good approach of health workers 4. Little expenses to deliver in this particular institution 5. Because I was told to have institutional delivery during ANC 6. Because I had previous caesarian section 7. I had previous difficult labor 8. Because I fear complications 9. Others, specify_______________________________ |  |
